# Characterization of a new apple luteovirus identified by high-throughput sequencing

**DOI:** 10.1186/s12985-018-0998-3

**Published:** 2018-05-15

**Authors:** Huawei Liu, Liping Wu, Ekaterina Nikolaeva, Kari Peter, Zongrang Liu, Dimitre Mollov, Mengji Cao, Ruhui Li

**Affiliations:** 1 USDA-ARS, National Germplasm Resources Laboratory, Bldg. 004/Rm 015, Beltsville, Maryland 20705 USA; 2 School of Life Science, Nanchang University, Nanchang, 330031 Jiangxi China; 3 Pennsylvania Department of Agriculture, Harrisburg, Pennsylvania 17110 USA; 4 Pennsylvania State University, Biglerville, Pennsylvania 17307 USA; 5 USDA-ARS, Appalachian Fruit Research Station, Kearneysville, West Virginia 25430 USA; 6 Citrus Research Institute, Southwest University, Chongqing, 400712 China

**Keywords:** Rapid apple decline, USA, Luterovirus, Genomic sequence

## Abstract

**Background:**

‘Rapid Apple Decline’ (RAD) is a newly emerging problem of young, dwarf apple trees in the Northeastern USA. The affected trees show trunk necrosis, cracking and canker before collapse in summer. In this study, we discovered and characterized a new luteovirus from apple trees in RAD-affected orchards using high-throughput sequencing (HTS) technology and subsequent Sanger sequencing.

**Methods:**

Illumina NextSeq sequencing was applied to total RNAs prepared from three diseased apple trees. Sequence reads were de novo assembled, and contigs were annotated by BLASTx. RT-PCR and 5′/3’ RACE sequencing were used to obtain the complete genome of a new virus. RT-PCR was used to detect the virus.

**Results:**

Three common apple viruses and a new luteovirus were identified from the diseased trees by HTS and RT-PCR. Sequence analyses of the complete genome of the new virus show that it is a new species of the genus *Luteovirus* in the family *Luteoviridae*. The virus is graft transmissible and detected by RT-PCR in apple trees in a couple of orchards.

**Conclusions:**

A new luteovirus and/or three known viruses were found to be associated with RAD. Molecular characterization of the new luteovirus provides important information for further investigation of its distribution and etiological role.

**Electronic supplementary material:**

The online version of this article (10.1186/s12985-018-0998-3) contains supplementary material, which is available to authorized users.

## Background

Apple (*Malus domestica* L.) is the most widely cultivated fruit crop worldwide [1]. The U.S. is the world’s second-largest producer of apple with a wholesale value of $4 billion (https://www.usapple.org/all-about-apples/apple-industry-statistics/). Apple is propagated by grafting, budding and layering. The careless selection of infected materials for the propagation allows the accumulation of virus/viruses in apple trees and dissemination of viruses between trees, orchards and regions. At least ten viruses and four viroids have been reported to infect apple trees, causing many types of diseases that reduce fruit quality and yield [2]. Among the most commonly reported viruses are apple stem pitting virus (ASPV), apple stem grooving virus (ASGV) and apple chlorotic leaf spot virus (ACLSV), all species of the family *Betaflexiviridae*. These viruses are ubiquitous and frequently occur as mixed infections. They are commonly called latent viruses because they usually do not induce obvious symptoms in most cultivars used in production, although yield reductions have been reported [3].

**Fig. 1:**
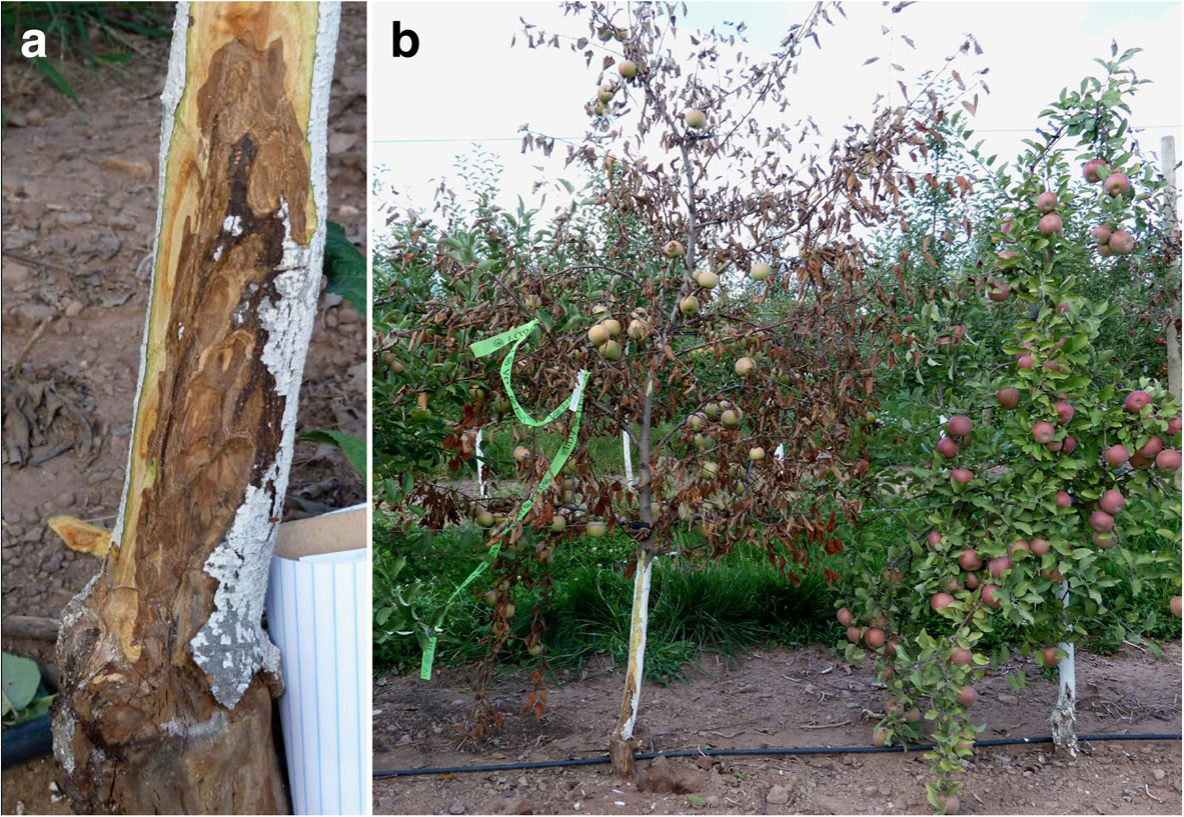
Symptoms of rapid apple decline. Necrosis of the graft union (**a**) and rapid collapse (**b**) of an apple cv. Fuji tree grafted on M9 rootstock

For the last several years, an unusual problem of young, apple trees growing of dwarfing rootstock in the northeastern U.S. has been observed (https://extension.psu.edu/apple-disease-rapid-apple-decline-rad-or-sudden-apple-decline-sad). The problem has been named ‘Rapid Apple Decline’ (RAD) or ‘Sudden Apple Decline’ due to the rapid or sudden death of apple trees after the first appearance of symptoms (Fig. 1). Several scion cultivars start to decline after grafting onto the M9 rootstock. The affected trees usually exhibit cankers and cracks on the rootstock and/or scion trunks. Necrosis begins at the graft union and proceeds up the trunks. The leaves of some affected trees begin to look yellow and then redden, and within two weeks the trees collapse from late July through September.

The involvment of fire blight and other pathogens [phytophthora and tomato ringspot virus (ToRSV)], herbicide damage, winter and drought injuries in RAD has been largely ruled out through rigorous observations and/or testing. This prompted us to use high-throughput sequencing (HTS) to further investigate possible causal agent(s) of RAD.

HTS combined with bioinformatic analyses is becoming a routine technology in the field of plant virology for the discovery of many new and emerging viruses, detection of known viruses and investigation of viral genetic diversity and evolution [4]. In this study, Illumina RNA sequencing technology was used to identify the pathogens potentially associated with RAD, including a new luteovirus tentatively named apple luteovirus 1.

## Methods

### Sample collections and preparations

In June 2016, samples of branches were collected from six RAD symptomatic apple trees (Table 1; PA2, PA4, PA5, PA7, PA8 and PA9) in a 5-year-old orchard at the Pennsylvania State University Fruit Research and Extension Center (PSU-FREC). The apple cultivar was Crimson Crip grafted on M9 rootstock. Two samples, one from scion and another from rootstock suckers, were collected from each tree. For HTS analysis, total nucleic acids were extracted from leaf, petiole and bark tissues by a CTAB method [5] and used for total RNA isolation by RNeasy® Plant Mini Kit (Qiagen, Germantown, MD). TNA by the CTAB method were used for the detection of all four viruses.

**Table 1:** Cultivars, symptoms and viruses of apple trees in a research block

Sample^a^	Cultivar^b^	Symptoms^c^	Virus^d^			
			ALV 1	ACLSV	ASGV	ASPV
PA2	Crimson Crisp	Trunk cracking, leaf yellowing	+	+	+	+
PA4	Crimson Crisp	Trunk cracking, leaf yellowing	+	–	+	+
PA5	Crimson Crisp	Trunk cracking, leaf yellowing	+	+	+	+
PA7	Crimson Crisp	Trunk cracking, leaf yellowing	+	–	+	–
PA8	Crimson Crisp	Trunk cracking, leaf yellowing	+	–	–	+
PA9	Crimson Crisp	Trunk cracking, leaf yellowing	+	–	–	–
PA11	Crimson Crisp	NS	+	+	+	+
PA12	Crimson Crisp	NS	+	+	+	+
PA13	Crimson Crisp	Bark cracking	+	+	+	+
PA14	Crimson Crisp	NS	+	+	+	+
PA15	Crimson Crisp	Upper branch browning	+	+	–	+
PA16	Crimson Crisp	NS	+	+	+	+
PA17	Crimson Crisp	Leaf distortion, tree dying	+	+	+	+
PA18	Fuji	Trunk cracking, leaf curl	+	–	+	–
PA19	Fuji	NS	–	–	–	–
PA20	Gala	Trunk cracking, small leaves	+	+	+	–
PA21	Fuji	NS	+	–	–	–
PA22	Gala	NS	+	+	–	+
PA23	Golden Delicious	Trunk cracking, leaf curl	+	–	+	–
PA24	Golden Delicious	NS	+	–	–	–

^a^Samples PA2–9 were collected in June 2016

^b^All cultivars were grafted on M9 rootstock

^c^NS mean that there were no obvious symptoms

^c^*ALV 1* Apple luteovirus 1, *ACLSV* Apple chlorotic leaf spot virus, *ASGV* Apple stem grooving virus, *ASPV* Apple stem pitting virus

### High-throughput sequencing and analyses

Total RNAs of three pooled samples, CPAR (all 6 rootstock samples), CPAS1 (scion samples of PA2, PA4 and PA5) and CPAS2 (scion samples of PA7–9), were processed at SeqMatic (Fremont, CA). Plant ribosomal RNAs (rRNA) were removed from total RNAs using Illumina Ribo-Zero rRNA Removal Kit for cDNA library construction. The samples were sequenced on the Illumina NextSeq sequencing platform with 15-sample bar-coded multiplexing.

Analyses of total sequence reads were performed using the CLC Genomics Workbench 9.5.2 platform (https://www.qiagenbioinformatics.com/). The raw reads were filtered to remove the failed reads, and qualified reads were assembled de novo into contigs with a cut-off of 150-nt. Contigs were annotated by BLASTx comparisons to Viruses_NR and Viroids databases downloaded from NCBI GenBank databases.

### Validation of the viruses and genome determination of a new luteovirus

To verify the presence of a new luteovirus in the six samples used in HTS, RT-PCR using primers AluDetF6/R6 (Additional file 1) were used. The three latent viruses were also detected by RT-PCR using virus-specific primers (Additional file 1) designed based on alignments of their genomic sequences available in GenBank. The RT-PCR was performed using the SuperScript™ III One-Step RT-PCR System (Invitrogen, Carlsbad, CA, USA) in a 20-μl reaction containing 1 μl of TNAs, 1.0 μl of each primer (5 μM), 10 μl of 2× Reaction Mix, 0.4 μl of Enzyme Mix and 6.6 μl of water. The thermal cycling conditions for RT-PCR were 1 cycle of 50 °C for 30 min and 94 °C for 2 min, 35 cycles of 94 °C for 30 s, 55–60 °C (varied according to the primer pairs) for 1 min and 68 °C for 40 s and one final extension at 68 °C for 5 min.

To obtain the complete genomic sequence of the new luteovirus, primers were designed based on the contig sequences with similarity to several luteroviruses (Additional file 1). The TNAs of the PA8 sample (Table 1) was used as template in the RT-PCR. The 5′-end sequence was obtained by a 5’RACE System Kit (Invitrogen). The 3′-end sequence was determined by a First Choice RLM-RACE Kit (Invitrogen) after polyadenylation of the RNAs using poly(A) polymerase (New England BioLabs, Ipswich, MA). All amplicons were cloned into pGEM-T Easy Vector (Promega, Madison, WV), and plasmid DNAs isolated from overnight cultures were sequenced (MCLAB, San Francisco, CA).

### Genomic sequence analysis of the luteovirus

The sequences were assembled and analyzed by the CLC Genomics Workbench. Open reading frames (ORFs) were predicted using the Open Reading Frame Finder at https://www.ncbi.nlm.nih.gov/orffinder/. Multiple alignments of genomic sequences and deduced amino acid sequences of individual viral genes were performed by the neighbor-joining algorithm as implemented in ClustalW, and the resulting alignments were analyzed using MEGA7 [6]. Recombination analysis was carried out using RDP 4.83 package [7].

### Graft transmission of the luteovirus

To verify the luteovirus sequences are associated with graft-transmissible agents, dormant buds of each of four selected trees from the PSU block (Table 1; PA13, PA14, PA18 and PA21) were grafted onto seven apple seedling trees. All seedlings tested negative for the luteovirus and three latent viruses. PA21 is infected with the luteovirus alone, but PA13, PA14 and PA18 are mix infected with at least one latent virus. Leaves were collected from new shoots of each of the inoculated seedlings at one, five and seven months after grafting. TNA were extracted from leaf and petiole tissue and used as template in by RT-PCR as described above. An uninoculated apple seedling was used as negative control.

### Additional detection of the luteovirus

Eighty samples were collected from both RAD symptomatic and symptomless apple trees from PSU-FREC and USDA-ARS Appalachian Fruit Research Station (AFRS) in West Virginia (Table 2). The FREC trees in Pennsylvania were grafted on M9 rootstock, whereas the AFRS trees were grafted on EMLA 7 rootstock. A symptomatic tree (PA14) infected with the luteovirus and three latent viruses and an apple seedling were used as controls. TNAs prepared from leaves, petioles and bark were used as templates in RT-PCR, as described above. Viral amplicons of selective samples of each location were cloned and sequenced by Sanger sequencing.

**Table 2:** Apple samples used for detection of apple luteovirus^a^

Location^a^	Collection date	Cultivar^b^	Number of samples	Infection rate
PSU-FREC	09/16/2014	Fuji	12	11 (91.6%)
Pennsylvania		Gala	12	2 (16.7%)
		Gold Delicious	12	2 (16.7%)
AFRS	01/31/2017	Crimson Crisp	13	4 (30.7%)
West Virginia		Fuji	11	1 (9.1%)
		Hampshire	10	0
		Snapp Stayman	10	1 (10.0%)
Total			80	21 (26.3%)

^a^*PSU-FREC* Pennsylvania State University-Fruit Research and Extension Center, *AFRS* Appalachian Fruit Research Station

^b^Cultivars in the PSU-FREC orchard were grafted on M9 rootstock, and cultivars in the AFRS orchard were grafted on EMLA 7 rootstock

## Results

### Virus identification by high-throughput sequencing

Total reads of 27,727,559 (CPAR), 28,817,295 (CPAS1) and 30,614,167 (CPAS2) were obtained after removing the failed reads. Assembly de novo of the reads generated a total of 59,415 (CPAR), 60,992 (CPAS1) and 65,115 (CPAS2) contigs larger than 150 nt. Blastx searches against the Viruses_NR database revealed contigs with amino acid (aa) sequence identities of 29–76% to peach associated luteovirus (PaLV), cherry associated luteovirus (ChaLV) and rose spring dwarf associated virus (RSDaV) of the genus *Luteovirus* in the family *Luteoviridae* from CPAR (2 contigs), CPAS1 (6 contigs) and CPAS2 (2 contigs), respectively. Approximately 335,446 (86×), 48,908 (12×) and 778,086 (199×) reads were mapped to contig CPAS1–8, the longest luteovirus contig, supporting the presence of the virus in the three samples. Multiple contigs with identities of 83–99% to ACLSV, ASGV and ASPV were also identified from all three samples, respectively, but the data are not presented here. No viroid was detected from any of the HTS samples.

### RT-PCR detection the viruses in orchard samples

The luteovirus was detected in all six HTS samples (6/6, 100%), whereas ACLSV (2/6, 33%), ASGV (4/6, 67%) and ASPV (4/6, 67%) were only found in some of these trees (Additional file 2). PA9 was infected with the luteovirus alone, whereas five other trees were infected with at least one latent virus. Fourteen more samples consisting of four different cultivars were collected from the same PSU-FREC orchard and tested by RT-PCR. The luteovirus was detected in 13 of them (93%) (Table 1). The latent viruses were again detected from fewer samples (ACLSV 64%, ASGV 64% and ASPV 57%).

To expand the testing for the luteovirus, a total of 80 additional samples were collected from the PSU-FREC and AFRS orchards (Table 2). Some trees in the PSU-FREC orchard showed the disease symptoms, whereas those in the AFRS orchard did not have obvious symptoms. Results of RT-PCR showed that majority of cv. Fuji trees (11/12) and a small number of cv. Gala (2/12) and Gold Delicious (2/12) trees in the PSU-FREC orchard were infected with ALV-1, but the infection rates were much lower for the cultivars in the AFRS orchard.

### Graft transmission

The graft inoculated apple seedlings did not display obvious symptoms nine months after inoculation. Infections of the luteovirus and latent viruses were confirmed by RT-PCR using specific primers, respectively (Additional file 1). A weak amplification was obtained from the PA21 seedlings one month after inoculation (data not shown), and all four samples tested positive five months after inoculation (Additional file 3).

### Complete genome of the luteovirus

Complete genome of the new virus, with the proposed name apple luteovirus 1 (ALV-1), is 6001 nucleotides (nt) in size (GenBank no. MF120198), encoding ten open reading frames (ORFs). The genome starts with a conserved element^1^GTGAUU^6^ (underlined nt is different from other species of *Luteovirus*) and contains all cis-acting elements of the luteoviruses [8, 9]. The conserved ^1364^GGAUUUUUAGAGGGGCU^1380^ and ^1392^CCGGCUUUGAAUCCCCUUU^1410^ known to be responsible for the − 1 ribosomal frameshift are located at the junction of ORF1 and ORF2. A tract of ten tandem CCXXXX (X is any base) repeats that is required for the ORF3 stop codon readthrough starts at nt 3695. The 3′ terminal region contains all conserved elements [8] but the longest motif at the first stem-and-loop of the barley yellow dwarf virus-like element (BTE) is different as ^5503^GUA***CG***UCCUGGUA***G***AACAGG^5522^ (bolded and italicized nt represents inserted nt). These two insertions, ^5506^CG^5507^ and ^5516^G, are unique to ALV-1.

**Fig. 2:**
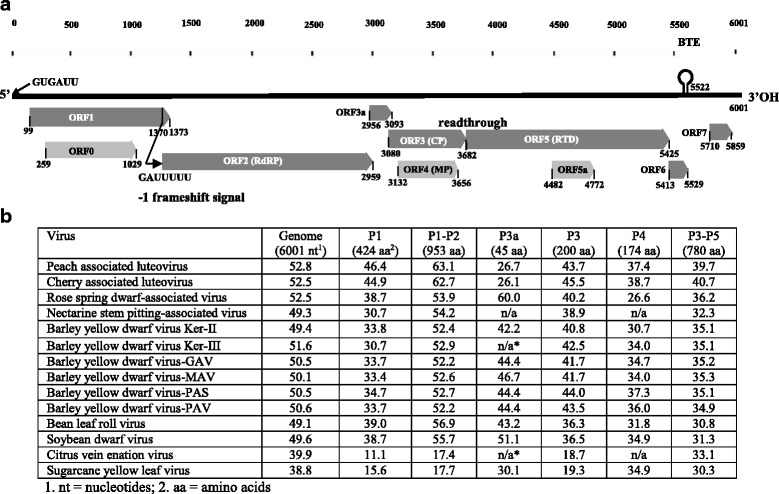
Genomic organization of apple luteovirus 1 (**a**) and its sequence identity percentages with other viruses of the genus *Luteovirus* and representative viruses of other genera in the family *Luteoviridae * (**b**). BTE, barley yellow dwarf virus-like translational element; n/a, data not available. The numbers below each open reading frame (ORF) box indicate the positions of the start and stop codons of each ORF, respectively

The arrangement and structure of six of these ORFs (ORF1, ORF2, ORF3, ORF3a, ORF4 and ORF5) resembles that of other luteoviruses (Fig. 2a) [8, 10–14]. The ORF1 and ORF2 encode putative P1 and P1-P2 fusion proteins (by the − 1 frameshift translation), respectively, and together they form a putative replicase complex. The ORF3 encodes a putative coat protein (CP), and translation via read-through of its stop codon produces a putative P3-P5 fusion protein that might be involved in insect transmission. The ORF4 encodes a putative movement protein (MP). Like most luteoviruses, ALV-1 also encodes a small ORF3a (nt 2956–3093) that is essential for long distance movement [15]. ORF6 and ORF7 are only present in some luteoviruses [11, 13, 14], and ORF1a and ORF5a are unique to this virus. ORF1a embedded within ORF1 is in a similar position to ORF0 of species of the genus *Enamovirus* [16] but its gene product (264 aa residues) does not share any sequence similarity to the enomovirus ORF0. ORF5a within ORF5 encodes a putative protein of 96 aa residues. The putative proteins encoded by ORF1a and ORF5a have no sequence homology with any known proteins.

### Sequence comparisons and phylogentic analysis of the luteovirus

Comparisons of genomic and individual protein sequences among species in the family *Luteoviridae* confirmed that ALV-1 is most closely related to PaLV (Fig. 2b). The genome sequence identities between the two viruses is 53% at the nucleotide sequence level, which falls within range of 48–69% among the luteoviruses [17]. Sequence comparisons showed that the P1-P2 replicase was the most conserved (52–63%), whereas the P4 (MP) was the least conserved (27–39%) between ALV-1 and known luteoviruses at aa sequence level. Except for the P3a, ALV-1 had the highest aa sequence identity with PaLV or ChALV at the individual proteins. The P3a of ALV-1 was most closely related to soybean mosaic virus (SMV). According to the species demarcation criteria for the family *Luteoviridae* (≥ 10% difference in aa sequence of any gene) [17], ALV-1 should be a new species of the genus *Luteovirus*.

**Fig. 3:**
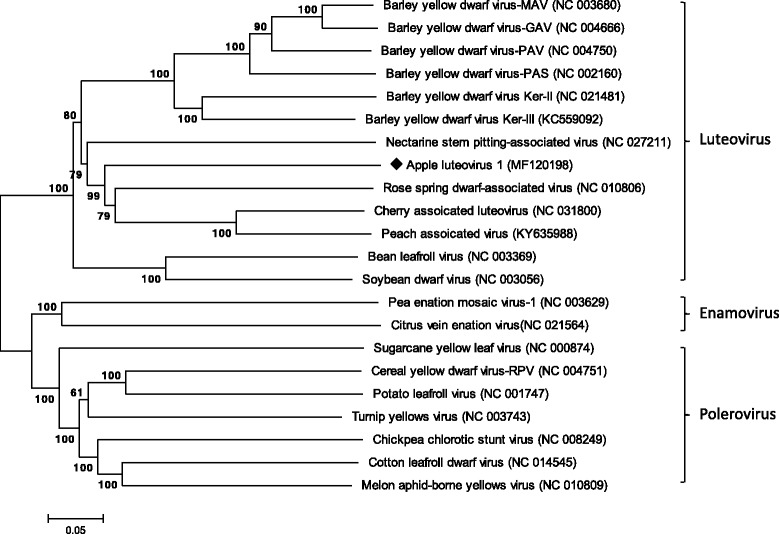
Neighbor-joining tree derived from complete genomic sequences of apple luteovirus 1 and representative members of the family *Luteoviridae*. Bootstrap analysis was applied using 1000 replicates. Solid diamond indicates the virus characterized in this study

Phylogenetic analyses conducted using the genomic sequences of ALV-1 and other luteoviruses placed the virus with ChALV, PaLV, RSDaV and nectarine stem pitting-associated virus (NSPaV) in a cluster distinct from that of BDYVs and two legume luteoviruses (Fig. 3). Topologies of phylogenetic trees changed slightly when the aa sequences of the P1-P2 and CP aa sequences were analyzed but close relationship of the four viruses were retained (data not shown). Analysis of the genomic sequences of 45 species of the family *Luteoviridae* by RDP4 did not detect any recombination breakpoints.

## Discussion

A novel luteovirus provisionally named apple luteovirus 1 and three common viruses (ACLSV, ASGV and ASPV) were identified by HTS of total RNA extracted from RAD-affected apple trees. BLAST search in pathogen databases identified several contigs showing similarities with members of the genus *Luteovirus* in the family *Luteoviridae*, particularly with four known luteoviruses (ChALV, NSPaV, PaLV and RSDaV) that infect woody plants [11–14]. The 6001-nt genomic sequence of ALV-1 is the largest genome of the known luteoviruses due to insertions in ORF1, ORF3 and ORF5. The arrangement and structure of the ALV-1 genome resembles those of other luteoviruses, containing six hallmark ORFs of the genus (Fig. 2a), encoding proteins involved in replication (P1 and P1-P2 fusion protein), virion assembly (CP), movement (P3a and P4) and aphid transmission (P3-P5 fusion protein) [9–11]. The ALV-1 genome also has several unique features. The 17-nt BTE motif conserved in the 3′ terminus of all known luteoviruses changes to ^5503^GUA***CG***UCCUGGUA***G***AACAGG^5522^ in ALV-1 due to two insertions (bolded and italicized bases), making it a unique motif among the luteoviruses. The ALV-1 genome also contains two additional ORFs (1a and 5a) that are not present in any known luteoviruses. Pairwise comparisons showed that ALV-1 has the highest genomic sequence identity (52.8%) with PaLV [14], indicating ALV-1 is a distinct species of the genus *Luteovirus*. Phylogenetic analysis also placed ALV-1 with the woody plant-infecting luteoviruses, suggesting that these viruses share a common ancestor.

Recombination plays an important role in evolution of plant RNA viruses. Both bean leafroll virus and SMV are recombinants occurred before the species separation [17]. It is a pervasive phenomenon among BYDV species and isolates [18]. However, recombination analysis did not reveal any recombination events in the ALV evolution.

Graft inoculation of apple seedlings demonstrated that ALV-1 is graft transmissible, and the virus could be spread by vegetative propagation of scions or rootstock or both. The source of this luteovirus is unknown, and subsequent study of rootstock and mother trees of different apple cultivars is necessary to determine the original infection.

Symptoms of RAD are very similar to apple union necrosis and decline (AUND) described in New York in early 1980s [19]. The eight apple cultivars propagated on MM106 rootstock showed graft union necrosis and tree decline. Similar diseases such as citrus tristeza quick decline disease (T-QD) [20] and citrus sudden death (CSD) have described in citrus trees [21]. T-QD destroyed millions of sweet citrus trees (*Citrus sinensis* L. OSb.) propagated on sour citrus rootstock (*C. aurantium* L.) worldwide many years ago [19], and CSD has started to kill hundreds of thousands of sweet oranges propagated on Pangpur lime rootstock (*C. limonia* L. OSb.) in Brazil since 1999 [20]. T-QD is caused by citrus tristeza virus (CTV), and CSD has been associated with citrus sudden-death associated virus (CSDaV), a species of the genus *Marafivirus* in the family *Tymoviridae*. RAD is only observed on apple trees grafted on certain dwarf rootstock cultivars such as M9. Both T-QD and CSD also occurred only on sweet orange grafted on certain rootstock [20, 21]. T-QD was controlled by replacement of the sour orange rootstock with CTV-tolerant rootstock such as Rangpur lime and Volkamer lemon.When examined by RT-PCR assay, ALV-1 was detected by RT-PCR in trees from the RAD-affected orchard (Tables 1 and 2). However, not all ALV-1-infected trees showed trunk cracking and foliar yellowing (Table 1). Similar results were obtained from AUND-, T-QD- and CSD-affected orchards. In AUND-affected orchards, the majority (93%) of the symptomatic trees and a few asymptomatic trees tested positive for ToRSV, a virus not detected in the RAD-affected orchard [19]. CTV was detected from both symptomatic and asymptomatic trees [20]. Trunk sectioning of a few trees in the PSU-FREC orchard revealed that an asymptomatic tree also had necrosis near the graft union, and length of the necrosis was correlated with severity of RAD (data not shown). This study suggested the symptoms on the trunk develop gradually in affected trees. Citrus trees infected with CSDaV have an incubation period of at least two years before symptoms of sudden death are observed [21]. Similarly, apple trees may remain functional and healthy in early infection before appearance of the tree decline and trunk symptoms such cracks and cankers, which could be caused by secondary fungal/bacterial infection on the trees weaken by the virus infection. The ALV-1 titer may also vary in different parts of an infected tree and from season to season due to the phloem limitation of the luteovirus, affecting the RT-PCR detection of ALV-1. Further study is needed to correlate the symptom development and ALV-1 infection on asymptomatic apple trees in orchards and apple seedlings inoculated with singly infected sources such as PA21.

ALV-1 was detected in both PSU-FREC and AFRS orchards, but RAD was only observed in the PSU-FREC orchard. Although most cultivars in the two orchards are different and have different infection rates, ALV-1 is detected in the common cv. Fuji trees. However, RAD was not observed in the Fuji tree block in the AFRS orchard where EMLA 7 rootstock was used. Therefore, M9 may be susceptible to ALV-1. Occurrence of AUND, T-QD and CSD had been associated with trees grafted on virus-susceptible rootstocks, respectively [19–21]. Replacement of the sour orange rootstock with CTV-tolerant rootstocks controlled T-QD [20]. Further investigation of ALV-1 infection in different rootstocks and scion-rootstock combinations is necessary to understand the role of susceptible rootstocks in RAD.

Nothing is known about the vector transmission of ALV-1. Salem et al. [22] reported that RSDaV is transmitted by rose-grass aphid (*Metapolophium dirhodum* Walker) and yellow rose aphid (*Rhodobium porosum* Sanderson). The aphid transmission of ChALV, NSPaV and PaLV has not been studied yet. Although aphids were observed in some RAD-affected orchards, their role in spreading ALV-1 in orchards needs to be defined.

Koch’s postulates have not yet been fulfilled so we cannot yet conclude that ALV-1 is a causal agent of RAD. The ALV-1 infection in the PSU-FREC orchard affected with RAD is very common, with 93% of the trees tested positive for ALV-1 and 57–64% tested positive for the three apple latent viruses (Table 1). Mix infection of the viruses in the RAD-affected orchard makes it difficult to associate ALV-1 with RAD. We are conducting bud grafting inoculation of ALV-1 on few apple cultivars grafted on M9 and other rootstocks to determine the role of ALV-1 in RAD.

The identification of a novel luteovirus in apple broadens the host range of the luteoviruses. Detecting ALV-1 from the RAD-affected trees justifies investigations of the role of this virus in the etiology of this newly emerging problem. The genomic sequence of ALV-1 obtained in this study enables the development of a specific RT-PCR for the rapid detection of the virus. This is important to study the distribution, transmission and pathogenicity of ALV-1.

## Conclusion

This paper reports the identification of a new luteovirus and/or three known viruses associated with RAD in Northeast USA. The genome of apple luteovirus 1 was obtained, and the virus was proven to be transmitted by grafting. These data provide important information for further investigation of the role of ALV-1 in RAD.

## Additional files

Additional file 1: Primers used in this study. (DOCX 13 kb)

Additional file 2: Virus detection by RT-PCR in RAD-affected apple trees used for high-throughput sequencing. The four viruses are apple luteovirus 1 (ALV-1), apple chlorotic leaf spot virus (ACLSV), apple stem grooving virus (ASGV) and apple stem pitting virus (ASPV), respectively. Lanes M) 1 kb plus DNA ladder, and W) water. Arrow indicate the DNA fragment with labeled size. (PPTX 226 kb)

Additional file 3: Verification of graft transmission of apple luteovirus 1 to apple seedlings by RT-PCR using primers AluDetF6/R6 (Additional file 1). Lanes M) 1 kb plus DNA ladder; 1–3) from PA13; 4–6) from PA14; 7–9) from PA18; 10–12) from PA21; 13) PA14; 14) water. Arrow indicate the DNA fragment with labeled size. (PPTX 157 kb)

## Abbreviations

ACLSV: Apple chlorotic leaf spot virus
ALV-1: Apple luteovirus 1
ASGV: Apple stem grooving virus
ASPV: Apple stem pitting virus
ChaLV: Cherry associated luteoviruses
HTS: High-throughput sequencing
NSPaV: Nectarine stem pitting-assoicated virus
nt: Nucleotide
ORF: Open reading frame
PaLV: Peach associated luteovirus
RAD: Rapid apple decline
RSDaV: Rose spring dwarf-associated virus
