# Supplementary material for: Characterization of a new apple luteovirus identified by high-throughput sequencing
Source: Virol J. 2018 May 15;15:85. doi: 10.1186/s12985-018-0998-3 (PMC5952423; doi:10.1186/s12985-018-0998-3)
Supplement: Supplementary file 1 — Primers used in this study. (DOCX 13 kb) [file 12985_2018_998_MOESM1_ESM.docx]

**Additional file 1. Primers used in this study**

| Name^1^ | Sequence (5’ to 3’) | Size of amplicon |
| --- | --- | --- |
| ACLSVF10a | TGCTGGGGTGAAAAGCTCCA | 1111 bp |
| ACLSVR10 | AGGATTGCCCCTGTCTTTCCATG |  |
| ASGV3 | AGAGAGGATTTAGGTCCCTCTC | 768 bp |
| ASGV6 | ACGACTCCTAACCCTCCAGTTC |  |
| ASPV1 | ATGTCTGGAACCTCATGCTGCAA | 370 bp |
| ASPV2 | TTGGGATCAACTTTACTAAAAAGCATAA |  |
| ALuDetF6 | AGCCAATGATTGTATTCGACGTG | 478 bp |
| ALuDetR6 | AGCTCTCTTCTAATGTGCGGAAC |  |
| ALu-5Tend2 | TCGTCCATGTAACCGTAACAG | 274 bp |
| ALu-5Tend1 | TCCTCGTCTTGGAACTTGTTC | 604 bp |
| ALuF1 | AGTGTGAAACCAAGCCCGTCAAG | 858 bp |
| ALuR1 | TGTGACACAGCTGGTCCGTCTTG |  |
| ALuF2 | AGAGAGTCGCCTTTACGGAGGAC | 1086 bp |
| ALuR2 | ACGTCAGCATCTTCCTTGCATAC |  |
| ALuF3 | ACCGTAGCGCAATTAGTCTCCAC | 1003 bp |
| ALuR3 | AGCTCTCAGATACCTTCTTGTAG |  |
| ALuF4 | AGAATGGTTCGTCGTCCTGATTC | 694 bp |
| ALuR4 | TCTTCGTCTTCGAGGCCTTCCTG |  |
| ALuF5 | TCGTGCGTAGACGTCAGCCAG | 1130 bp |
| ALuR5 | TCTCTCTACAACCTGACCGTC |  |
| ALuF6 | AGCCAATGATTGTATTCGACGTG | 1203 bp |
| ALuR6 | CAGTGATTGTCCGAGTTGATGCA |  |
| ALuF7 | AGCGTGATGGAGCTAACACC | 1186 bp |
| ALuR7^2^ | CCGACTGTAACCGCAATCGG |  |
| ALu-3Tend2 | CAATACAAGGGGAAAGCATCCA | 636 bp |
| ALu-3Tend1 | TGTACCCACCTTACCTAGTAGC | 575 bp |

1. ACLSV = apple chlorotic leaf spot virus; ASGV = apple stem grooving virus;

ASPV = apple stem pitting virus; ALu = apple luteovirus 1
