# Supplementary material for: Characterization of a new apple luteovirus identified by high-throughput sequencing
Source: Virol J. 2018 May 15;15:85. doi: 10.1186/s12985-018-0998-3 (PMC5952423; doi:10.1186/s12985-018-0998-3)
Supplement: Supplementary file 2 — Virus detection by RT-PCR in RAD-affected apple trees used for high-throughput sequencing. The four viruses are apple luteovirus 1 (ALV-1), apple chlorotic leaf spot virus (ACLSV), apple stem grooving virus (ASGV) and apple stem pitting virus (ASPV), respectively. Lanes M) 1 kb plus DNA ladder, and W) water. Arrow indicate the DNA fragment with labeled size. (PPTX 226 kb) [file 12985_2018_998_MOESM2_ESM.pptx]

## Slide 1
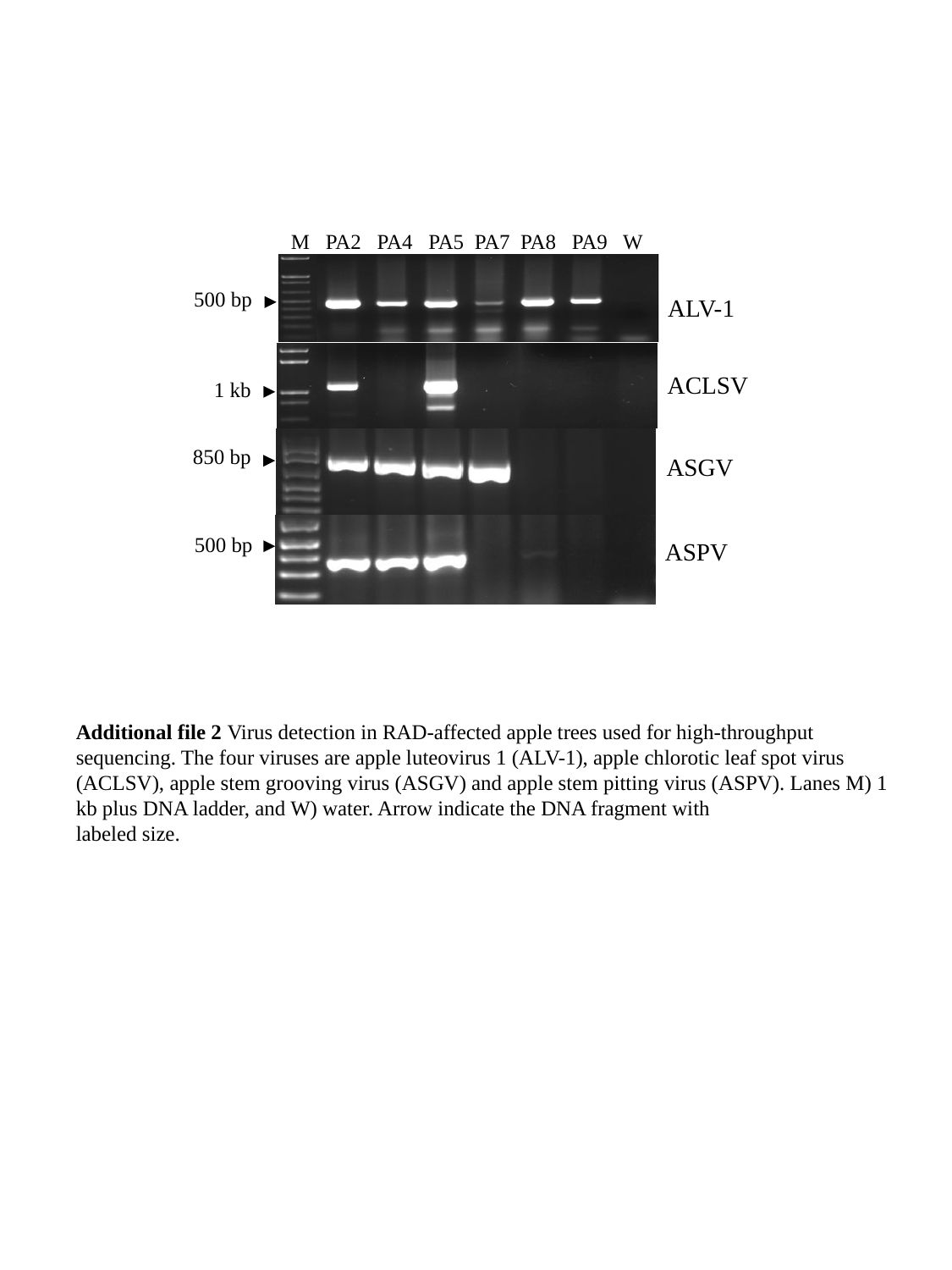

M PA2 PA4 PA5 PA7 PA8 PA9 W
500 bp
ALV-1
ACLSV
1 kb
850 bp
ASGV
500 bp
ASPV
Additional file 2 Virus detection in RAD-affected apple trees used for high-throughput sequencing. The four viruses are apple luteovirus 1 (ALV-1), apple chlorotic leaf spot virus (ACLSV), apple stem grooving virus (ASGV) and apple stem pitting virus (ASPV). Lanes M) 1 kb plus DNA ladder, and W) water. Arrow indicate the DNA fragment with
labeled size.
