# Supplementary material for: Characterization of a new apple luteovirus identified by high-throughput sequencing
Source: Virol J. 2018 May 15;15:85. doi: 10.1186/s12985-018-0998-3 (PMC5952423; doi:10.1186/s12985-018-0998-3)
Supplement: Supplementary file 3 — Verification of graft transmission of apple luteovirus 1 to apple seedlings by RT-PCR using primers AluDetF6/R6 (Additional file 1). Lanes M) 1 kb plus DNA ladder; 1–3) from PA13; 4–6) from PA14; 7–9) from PA18; 10–12) from PA21; 13) PA14; 14) water. Arrow indicate the DNA fragment with labeled size. (PPTX 157 kb) [file 12985_2018_998_MOESM3_ESM.pptx]

## Slide 1
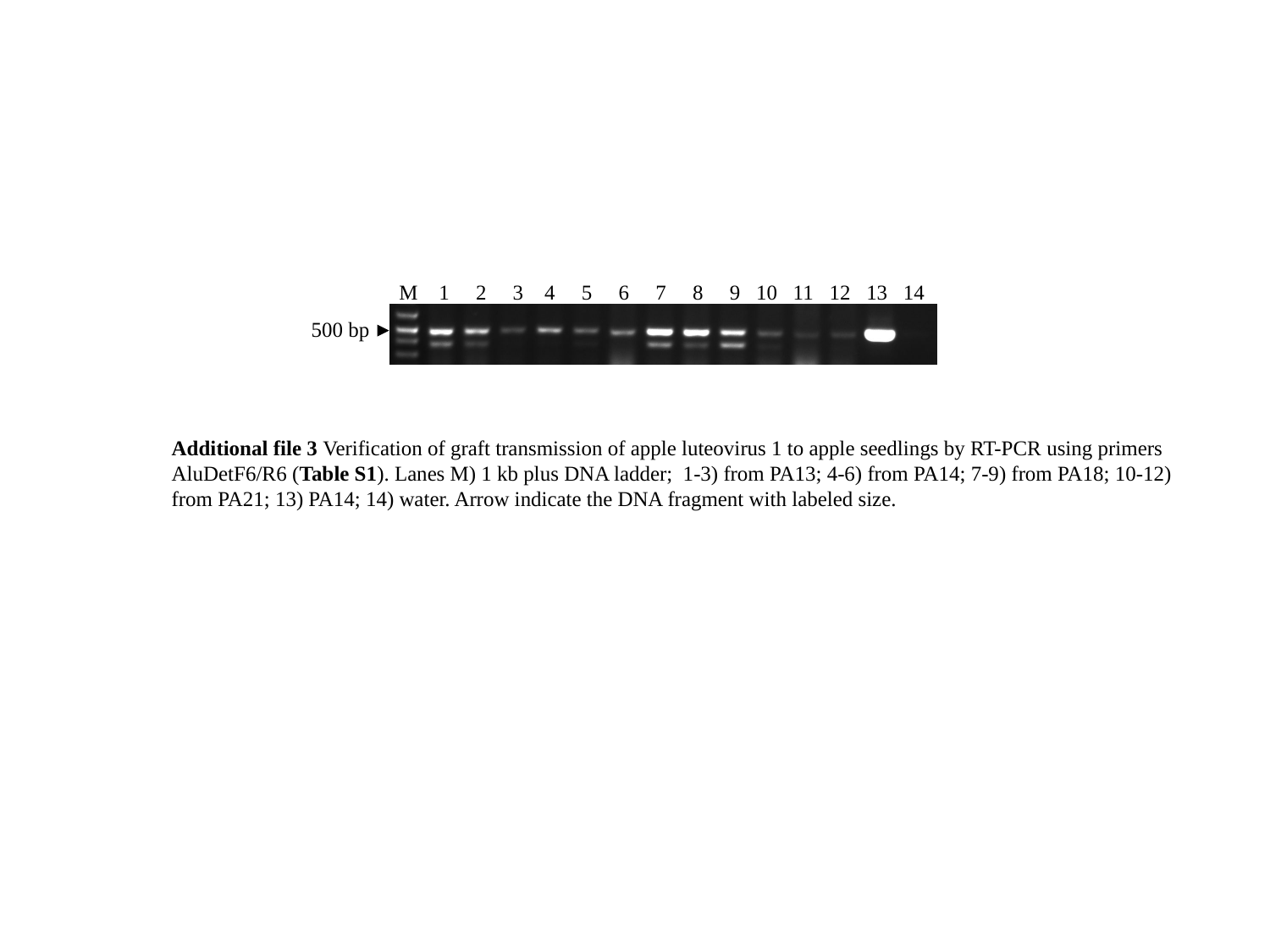

M 1 2 3 4 5 6 7 8 9 10 11 12 13 14
500 bp
Additional file 3 Verification of graft transmission of apple luteovirus 1 to apple seedlings by RT-PCR using primers AluDetF6/R6 (Table S1). Lanes M) 1 kb plus DNA ladder; 1-3) from PA13; 4-6) from PA14; 7-9) from PA18; 10-12) from PA21; 13) PA14; 14) water. Arrow indicate the DNA fragment with labeled size.
